# Structural impact of synonymous mutations in six SARS-CoV-2 Variants of Concern

**DOI:** 10.1371/journal.pone.0325858

**Published:** 2025-07-01

**Authors:** Alison Ziesel, Hosna Jabbari

**Affiliations:** Department of Biomedical Engineering, University of Alberta, Edmonton, Alberta, Canada; Shahid Beheshti University of Medical Sciences School of Medicine, IRAN, ISLAMIC REPUBLIC OF

## Abstract

SARS-CoV-2 continues to spread and infect people worldwide. While most effort into characterizing variants of this virus have focused on non-synonymous changes, accumulation of synonymous mutations in different viral variants has also occurred. Here we characterize six Variants of Concern in terms of their mutational content, and make predictions regarding the impact of those mutations on potential genomic RNA secondary structure and stability. Our hypothesis is that if non-protein changing, yet RNA structure-changing mutations impact viral fitness by imposing deleterious change to predicted RNA structure, we would expect to those mutations to be less abundant, while if those synonymous mutations do not impact viral fitness through influence of RNA structure, we would see them more frequently than non-synonymous mutations. We find that synonymous mutations typically have no or modest impact to RNA secondary structure. As synonymous mutations are free from the selective pressure imposed on protein-altering mutations, the impact of synonymous mutations is largely limited to RNA secondary structure considerations. The absence of major, structure-altering synonymous mutations emphasize the importance of RNA structure, including within coding regions, to viral fitness. Synonymous mutations should be included in the characterization of emerging RNA viruses as these mutations may confer effects to viral fitness via RNA secondary structural modifications.

## Introduction

SARS-CoV-2, the causative agent of the COVID-19 pandemic, is a single stranded RNA virus belonging to the clade Betacoronaviridae [1]. Its original host species may have been horseshoe bats, as the most closely related viruses to SARS-CoV-2 infect those species [2,3]. SARS-CoV-2 is also closely related to SARS-CoV, the causative agent of the SARS outbreak of 2002-2004 and shares a common receptor with SARS-CoV, the angiotensin converting enzyme 2 (ACE2) protein [4–6].

As the pandemic has proceeded, a number of novel Variants of Concern (VoCs) have arisen in different geographic regions over time. The World Health Organization (WHO) includes the following features as characteristic of a Variant of Concern [7]:

- exhibits genetic changes associated with increased transmissibility, virulence, immune evasion, susceptibility to therapeutics/detectability
- observed to have a growth advantage over other circulating variants in more than one WHO region
- detrimental changes in clinical disease severity, in epidemiology impacting health systems, or decrease in vaccine effectiveness

**Fig 1:**
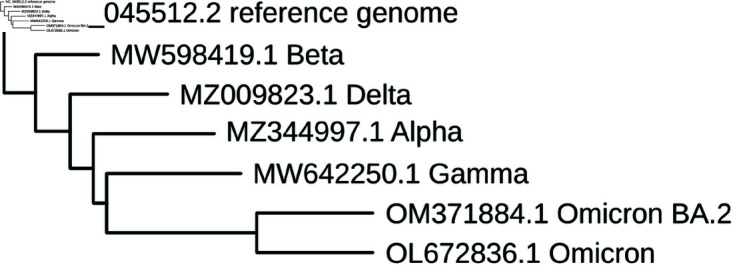
Phylogenetic tree indicating the relationship between six major VoCs and the reference genome for SARS-CoV-2.

In the case of SARS-CoV-2, VoCs are typically described in terms of their characteristic protein-altering, i.e. non-synonymous mutations, particularly protein-changing mutations of the Spike protein. Information on non-coding sequences (such as untranslated regions or intergenic regions) or synonymous mutations within coding regions is typically not provided. Here we summarize six recent VoCs in terms of their described mutations. An excellent review of the major VoCs prior to the summer of 2021 is available [8]. These six VoCs were chosen for their epidemiological significance and in the case of Omicron BA.2, as it was the emerging subvariant at the time of data collection. Further, Omicron BA.2 represents the ancestral strain for a large number of currently circulating Omicron variants [9]. While the original Omicron is relevant for its emergence, BA.2 is particularly relevant as its descendant lineages carry many if not all of the mutations present in BA.2. A comprehensive overview of the characteristic non-synonymous mutations of all VoCs, Variants of Interest (VoIs), and sublineages of SARS-CoV-2 may be viewed at outbreak.info [10]. Fig 1 shows the phylogenetic relationship of six major VoCs, detailed below, with the reference genome sequence for SARS-CoV-2.

### Alpha

The first major VoC, Alpha or B.1.1.7, was first identified in the late summer of 2020 [11]. It is characterized primarily by its mutations N501Y, P618H and Δ69-70 of the Spike gene which enhance transmission and infectivity, may enhance cleavage of the Spike surface protein into its S1 and S2 components leading to enhanced viral entry, and may compensate for immune escape mutations respectively [11–19].

### Beta

The Beta or B.1.351 variant emerged in late 2020 and includes the Spike mutations K417N, E484K and N501Y which each enhance binding of Spike to its receptor ACE2 [20,21]. Both Alpha and Beta were found to have increased transmissibility relative to the original SARS-CoV-2 strain, with Beta being less transmissible than Alpha [20,22,23].

### Gamma

The Gamma or P.1 variant was first characterized in late 2020, and includes the Spike mutations N501Y, E484K and K417T; similarly Gamma was observed to be highly transmissible relative to the original strain of SARS-CoV-2, but less transmissible than Alpha [23,24]. As observed in the Alpha and Beta variants, the N501Y mutation of the Spike protein extends the host range of SARS-CoV-2 to include other mammals [25]. The Spike mutation H655Y also has been found to enhance protein cleavage, host-virus fusion, and transmission in an animal model [26].

### Delta

The Delta or B.1.617.2 variant exhibits the characteristic Spike mutations L452R, P681R, T478K and E484Q, which may facilitate antibody evasion, improve viral fusion with the host receptor, may enhance host receptor binding, and act as an escape mutation [27–31]. Delta exhibits both increased transmissibility as well as reduced susceptibility to monoclonal antibodies approved for emergency use by the American Food and Drug Administration (FDA) [32,33].

### Omicron

In November 2021, the Omicron or B.1.1.529 variant was designated as a VoC. Omicron and its subvariants are the most highly mutated VoC to date, with at least 32 mutations in the Spike protein including N501Y, N679K and P681H [34–36]. Omicron’s Spike protein is predicted to have a higher electrostatic surface potential than the original SARS-CoV-2 Spike, possibly leading to increased affinity or binding of the ACE2 host receptor protein [37]. Arising independently in this lineage, the mutation H655Y of the Spike protein is found to improve viral transmissibility, Spike protein cleavage and fusion with target cells, as was found in the Gamma VoC [26].

### Omicron BA.2

The closely related subvariant Omicron BA.2 was first identified in late 2021 and includes a different but overlapping spectrum of mutations with its parent Omicron strain [35]. *In vitro* work with ACE2-expressing 293T cells has demonstrated that Omicron BA.2 is more infectious than Omicron and non-Omicron, D614G-bearing SARS-CoV-2 variants, but is not significantly more prone to immune escape than Omicron or D614G-bearing SARS-CoV-2 variants in a study of monovalent or bivalent mRNA-vaccinated sera [38]. In particular, the Spike mutation L371F was found to increase both fusogenicity and pathogenicity of this variant, as well as grant resistance to certain monoclonal antibodies [39].

### SARS-CoV-2 genomic organization

As a single stranded RNA virus, SARS-CoV-2 and other coronaviruses are known to form RNA secondary structures including pseudoknots. RNA secondary structures are formed when RNA molecules form intra- or intermolecular base pairs, which generates discrete regions of double stranded RNA referred to as stems or helices. Regions of unpaired RNA nucleotides isolated by a stem are referred to as loops. Pseudoknots are formed when the bases within one isolated loop interact with other nucleotides elsewhere in the RNA sequence. Structural stability is quantified as minimum free energy (MFE); a lower MFE for a given RNA secondary structure is associated with an increased likelihood of its occurrence in a biological system. Studies in SARS-CoV, SARS-CoV-2, and related viruses including murine hepatic virus (MHV), bovine coronavirus (BCov) and others have indicated organized structure in the 5 ′ and 3′ untranslated regions (UTRs) [40–54] as well as within the frameshifting stimulatory element (FSE) [55–57].

The 5′ UTR was characterized largely on work done in MHV and it is predicted to contain five stem-loop (SL) structures, each of which play a role in the viral life cycle. These five stem-loops are believed to be involved in the formation of subgenomic RNAs (sgRNAs) and in the initiation of translation of ORF1ab [40–46]. SL1 is believed to be involved in a number of key viral functions including protection from Nsp1-mediated translational repression [47]. SL2 is implicated in the production of sgRNAs, and exhibits a conserved YUUGY pentanucleotide loop [41,42]. SL3 contains the transcription regulatory sequence leader element, and is predicted to unfold to expose that element for sgRNA synthesis [42,44]. SL4 consists of two stems separated by a bulge, and may act as a spacer element as its deletion is lethal in studies of MHV [48]. Finally, SL5 is a multibranched stem loop including three terminal stem loops named SL5a through SL5c, and includes the start codon for ORF1a, indicating that this structure must be unfolded to begin translation. The exact function of SL5 is not yet understood, although mutations of SL5a can impact viral replication efficiency [46].

**Fig 2:**
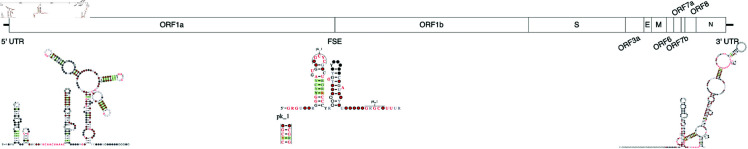
Predicted structures of the 5′ and 3′UTRs and the frameshifting stimulatory element (FSE) of SARS-CoV-2.

Similarly the 3′ UTR exhibits organized structure: shortly after the stop codon of the N gene, the 3′-most gene of the SARS-CoV-2 genome, the 3′ UTR bulged stem-loop structure (BSL) begins, consisting of four stems interspersed with three unpaired bulged subsequences and terminating in a loop [49,50]. The base of the BSL may alternately interact with an adjacent hairpin structure forming a pseudoknot, where either the pseudoknot or the base of the BSL may be formed, but not both simultaneously; it is thought that this arrangement functions as a molecular toggle switch, oscillating between states to regulate functions pertaining to viral replication [51,52]. The adjacent hairpin, known as P2, is part of a larger, poorly conserved region known as the hypervariable region (HVR). The 3′-most element of the SARS-CoV-2 genome is the *stem-loop II*-like motif element (s2m), which may play a role in host translation interference [53,54]. The structures and relative locations of the UTRs and FSE are depicted in Fig 2.

Internal to the SARS-CoV-2 genome and located at the junction between ORF1a and ORF1b, the FSE acts to establish the correct reading frame for ORF1b and all downstream genes by causing an actively translating ribosome to shift back by one nucleotide [55]. Structurally the FSE consists of a seven nucleotide ‘slippery site’, where the ribosome slippage occurs, followed by a five nucleotide spacer, and a pseudoknot that may act as a barrier to the proceeding ribosome, causing slippage within the slippery site approximately 15-30% of the time [55–57].

### Genomic structure prediction

Since the pandemic onset, work to characterize the secondary structure of SARS-CoV-2 both on a biochemical and a computational basis has been steady and continuous. Focusing on those computational approaches, predictions of SARS-CoV-2’s secondary structure were released by the Moss laboratory in early 2021, which included the details of their ScanFold-based computational pipeline identifying eight highly likely structures within the genome [58]. At nearly the same time the Pyle laboratory, using the SuperFold RNA secondary structure prediction utility, identified nearly 61% of the viral genome as being base paired and thus exhibiting secondary structure [59]. The Das laboratory, using a combination of CONTRAfold and RNAz identified 44 loci of predicted structure including components of the prototypical 5 ′untranslated region (UTR), frameshifting stimulatory element (FSE) and 3′ UTR [60,61]. The Mathews research group has published their findings using a recently developed iteration of their TurboFold algorithm, LinearTurboFold, and identified 50 structural elements, 26 of which were novel at the time of publication [62].

**Fig 3:**
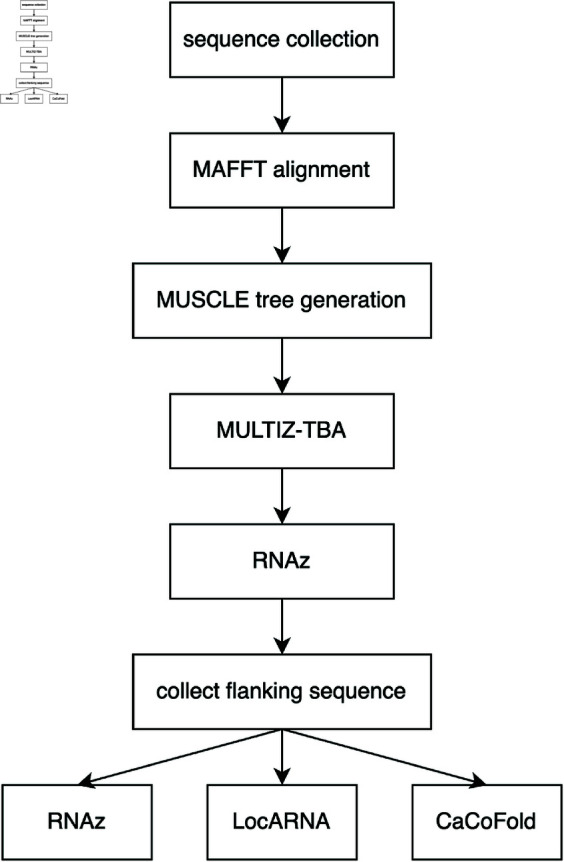
Flowchart describing the methods employed in our previous work.

Contemporaneously, we employed a combination of RNAz, LocARNA, and CaCoFold to identify secondary structure on the basis of conserved sequence and thermodynamic properties; we identified 40 regions of the genome as very favourable to the formation of secondary structure, with the workflow employed depicted in Fig 3 [63–66]; all parameters for this workflow are available in [66]. These three utilities were chosen for their strengths in identifying likely secondary structure due to thermodynamic properties, both in pseudoknot-free and pseudoknotted structures: RNAz is included in the well-regarded and popular ViennaRNA package, and combines both free energy and sequence conservation data to predict likelihood of forming an RNA secondary structure; LocARNA was developed as both a standalone program which performs simultaneous folding and alignment from a multiple sequenc alignment and as a companion program to RNAz, refining the output and improving the prediction of structure boundaries; CaCoFold uses stochastic context free grammar (SCFG) to predict RNA secondary structure, incorporating both positive and negative covariation data to determine likely structure. Using these three algorithms together allowed us to compare their predictions and identify commonly predicted regions of structure. Further, all three operate on multiple sequence alignment input, allowing us to leverage homology between coronaviruses to identify conserved regions of secondary structure. In that work we additionally compared our findings with a subset of previously published biochemical and computational findings regarding SARS-CoV-2 genome structure. We found that the previously characterized 5′and 3′UTRs but also intragenic regions of the genome were likely to generate structure. Additionally, we found that biochemically based approaches tended to obtain a more similar prediction to one another, while computational approaches varied depending on the algorithmic methods employed; any shortcomings of predictive approaches are addressed in prior work [66].

The goal of the research described here is twofold: to canvas major SARS-CoV-2 variants for synonymous mutations, and to characterize the impact on RNA secondary structure of both synonymous and nonsynonymous mutations on regions we previously predicted as likely to form organized structure. Our hypothesis is that if RNA secondary structure is important to viability of the SARS-CoV-2 virus, synonymous mutations will not lead to major impact on predicted genomic secondary structure. Work in microbial systems as well as human disease have indicated the significance of synonymous mutations beyond codon usage bias [67–69]. Conversely, if these predicted structures are not important to the viral life cycle and transmissibility, synonymous mutations will be as likely to cause severe changes as they are to cause negligible changes, indicating viral viability is preserved even when predicted structures are not. By measuring mutational rates, both synonymous and non-synonymous, and assessing their impact on predicted structure, we can test this hypothesis.

## Materials and methods

### Data

The reference sequence for SARS-CoV-2, NC_045512.2, was obtained through NCBI’s Nucleotide database [70]. All other sequences were obtained through GISAID’s EpiCoV database [9]. All sequences analyzed are available at https://doi.org/10.5281/zenodo.15505183. Comparisons were made to publicized mutations made available at outbreak.info [71]. outbreak.info reports protein-altering mutations present in at least 75% of the sequences they analyze for each VoC/VoI.

### Methods

#### Structure prediction.

We previously employed a computational pipeline to identify 40 regions likely to form secondary structure within the SARS-CoV-2 genome [63–66]. Fig 3 summarizes the workflow performed by our pipeline. In summary, thirteen coronavirus genomic sequences including SARS-CoV-2 were aligned to identify regions of the SARS-CoV-2 genome conserved in two or more additional coronavirus genomes. These conserved regions were then analyzed using RNAz, LocARNA, and CaCoFold to identify thermodynamically or grammatically stable secondary structures as well as covariant nucleotide positions within those structures.

#### VoC synonymous mutation identification.

For each of the VoCs Alpha, Beta, Gamma, Delta, and Omicron, at least 1000 whole genome samples were collected from GISAID on 14 January 2022. Samples for Omicron BA.2 were collected on 12 March 2022. At the time of collection, all available high coverage, complete Omicron sequences were collected for analysis. For all samples, the following criteria were imposed: the sample was flagged by GISAID as belonging to a given VoC; samples were marked as ‘high coverage’ with low coverage samples further excluded; samples must cover the entire genome of SARS-CoV-2; collection data (including date of collection, submission, and geographic location) was complete for each sample. The rationale for these restrictions was to gather only complete, high quality viral sequences for downstream analysis. Filtered results were sorted on descending sequence length to shuffle geographic and temporal origins of samples. Table 1 describes the number of samples collected for each VoC.

**Table 1:** Number of complete, high coverage viral genomes collected per Variant of Concern.

VoC	Number of samples
Alpha	1006
Beta	1009
Gamma	1012
Delta	1007
Omicron	5614
Omicron BA.2	1023

**Fig 4:**
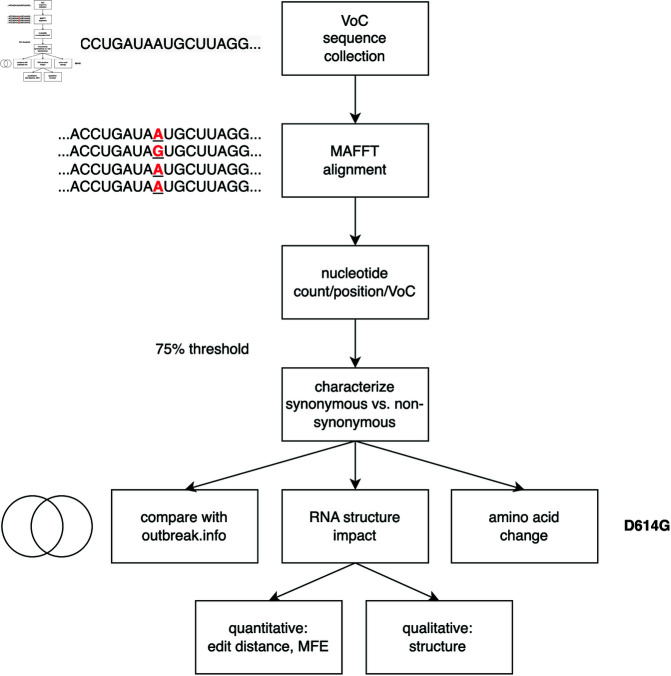
Flowchart describing the methods employed to analyze each Variant of Concern.

For each VoC collection, multiple sequence alignment was performed using MAFFT with default parameters; MAFFT is a fast and accurate alignment algorithm that scales well to large numbers of input sequences, making it especially appropriate for our applications [72]. We developed Python utilities to parse the resulting multiple sequence alignment at each nucleotide position as compared to the reference genome for SARS-CoV-2, NC_045512.2. This was done to identify all mutations relative to the reference genome for all viral sequences collected. For each position, each observed nucleotide variant was assessed for its frequency within the collected sequences for that VoC and mutations observed in at least 75% of the sequences for each VoC were retained; this cutoff was chosen to facilitate comparison with publicly available data offered by outbreak.info, a website specializing in curating SARS-CoV-2 variation data [71]. Next, a Python tool was developed to characterize each retained nucleotide level variant in terms of its impact on the SARS-CoV-2 proteome to identify synonymous and non-synonymous mutations. For each variant, we determined which codon and codon position that nucleotide variation occupied, identified the reference codon-specified amino acid present in the wild-type protein, and then determined the impact that the detected variation had on that codon. If a nucleotide variation did not alter the amino acid specified at that position, the variation was described as synonymous. Conversely, if a nucleotide variation did alter the amino acid specified at that position, the variation was classified as non-synonymous. A companion Python tool was written to convert publicized protein level changes to nucleotide level mutations; for each amino acid substitution, we determined which nucleotide must have mutated to cause a change from the reference to the mutant amino acid. Fig 4 characterizes the workflow of assessing each mutation for its characteristics including frequency and impact on protein sequence. In summary, mutations present in at least 75% of the samples analyzed were characterized in terms of their synonymous versus non-synonymous status, as well as their presence in the public data repository outbreak.info.

#### Structural impact.

All mutations present in at least 75% of the samples analyzed were then assessed for their impact on potential RNA secondary structure formed by the SARS-CoV-2 genome. In our previous research, we identified 40 regions of the viral genome that are most likely to form RNA secondary structures. This identification was based on sequence conservation, predicted thermodynamics, and nucleotide covariation. We then examined the mutations that occurred within these regions, as identified by this work [66]. Those mutations that were present within these 40 regions were then substituted into the reference genome sequence and the mutated subgenomic sequence was checked for structural consequences using RNAfold [73]. We elected to use RNAfold as it is a fast, inexpensive thermodynamic-based method that could quickly assess for any changes in predicted structure. The impact of each mutation on structure was assessed quantitatively by determining the MFE of the reference and mutant structures, and by measuring the Levenshtein string edit distance between the dot-bracket representations of the reference and mutant structures. Levenshtein string edit distance is a measure of the similarity of two sequences of symbols, and quantifies the number of changes needed to convert one sequence to the other. We implemented an algorithm to calculate Levenshtein distance in Python and quantified distances between wild-type and mutant dot bracket structural representations. Impact of mutated positions were assessed qualitatively through visualization using the secondary structure visualization utility VARNA; VARNA produces a high quality depiction of RNA secondary structure in a format easily assessed by the human eye [74]. Fig 4 provides a graphical overview of the methods outlined in this and the previous subsection.

#### Comparison with public data resources.

outbreak.info is a regularly updated, global repository of data on SARS-CoV-2 variants in circulation currently and historically [71]. Among the data resources available at their website are pages dedicated to describing VoCs/VoIs in terms of their protein-altering (non-synonymous) mutations. Non-synonymous mutations present in at least 75% of the sequences analyzed by outbreak.info are included as ‘Characteristic Mutations’ for a given variant. As mutations to UTRs and intergenic regions are not protein-altering, these mutations, along with synonymous mutations, are not included in this report. We compared mutations we identified at the same prevalence of 75% with those catalogued by outbreak.info to measure overlap in identification rates for non-synonymous mutations.

## Results

### Characterizations of VoCs

For each of the six VoCs considered, we describe the synonymous and non-synonymous mutations in both coding and non-coding regions detected in at least 75% of sequences. We report mutations by their nucleotide level change and position, and in the case of coding sequence mutations, in terms of their protein level change and position. The following sections detail our findings, organized by VoC. S1 Table summarizes our findings organized by mutation. We then compare our findings to a publicly available resource, and conclude by describing the impact of identified mutations on predicted secondary structure.

#### Alpha.

**Fig 5:**
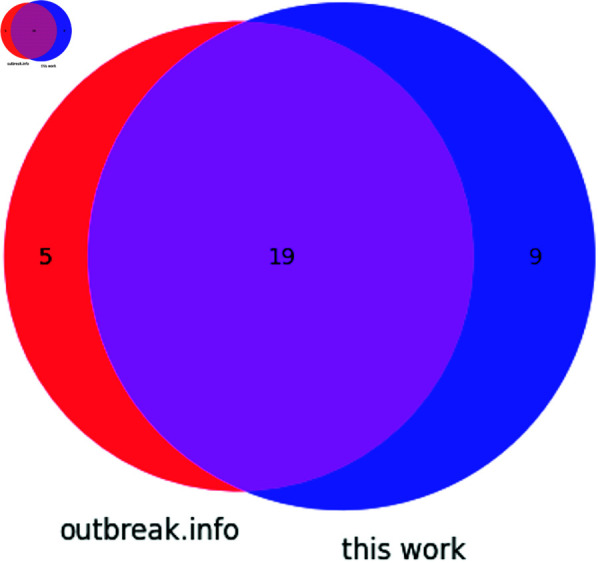
Venn diagram showing the distribution of mutations found in the Alpha variant according to outbreak.info and this work.

Seven synonymous and one non-coding mutation were detected in this analysis of SARS-CoV-2 Alpha sequences. Additionally, twenty non-synonymous mutations were identified. The overlap between outbreak.info’s reported non-synonymous mutations for Alpha and all mutations identified in this work is described in Fig 5. C241U, a mutation in the 5′ UTR’s SL5 was identified, as were the following synonymous mutations: in ORF1a, C913U (S216S), C3037U (F924F), C5986U (F1907F); in ORF1b, C14676U (P403P), C15279U (H604H), U16176C (T903T); and in N, G28882A (R203R).

#### Beta.

Two synonymous and two non-coding mutations were detected in Beta sequences. Additionally, 33 non-synonymous mutations were identified in this work.

**Fig 6:**
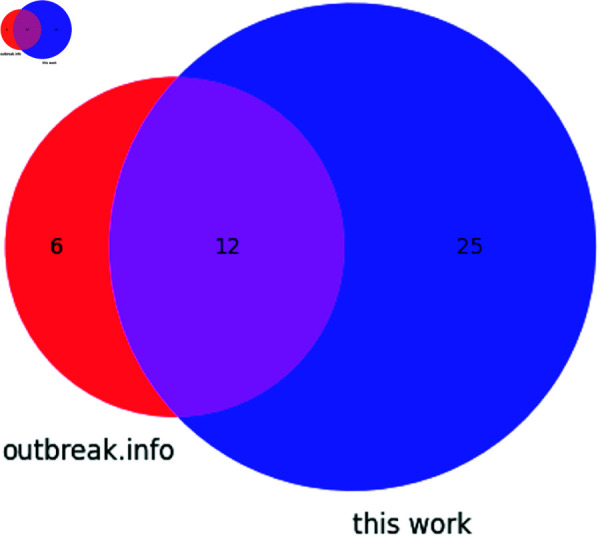
Venn diagram showing the distribution of mutations found in the Beta variant according to outbreak.info and this work.

There is slight disagreement as to the position of a deletion in the Spike gene: outbreak.info describes this as a deletion of amino acids 241 through 243, which would correspond to nucleotides 22282-22290, while in this work focused on nucleotide level mutation characterization, this deletion spans 22281 through 22289; both nucleotide deletions lead to the same amino acid sequence. We additionally identified a deletion in more than 75% of analyzed samples spanning nucleotides 11288 through 11296, or deletion of ORF1a (Nsp6) amino acids 3675-3677 (106-108; residues SGF). The overlap in identification between outbreak.info and this work is presented in Fig 6. Note that while adjacent deleted nucleotides present in more than 75% of analyzed genomes may represent a contiguous deletion, this cannot be confirmed and so the Venn diagram depicted in Fig 6 treats each nucleotide deletion as a separate mutation. G174U and C241U were identified in the 5′UTR, while C3037U (F924F of ORF1a) and C28253U (F120F of ORF8) were additionally identified.

#### Gamma.

**Fig 7:**
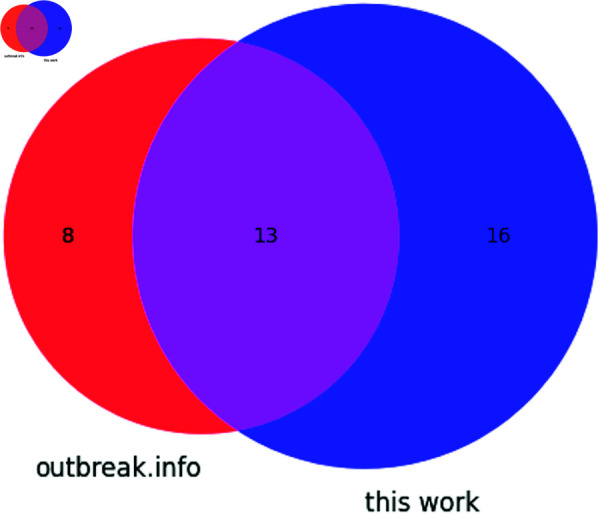
Venn diagram showing the distribution of mutations found in the Gamma variant according to outbreak.info and this work.

In the Gamma VoC of SARS-CoV-2, seven synonymous and five non-coding mutations were identified, along with 17 non-synonymous mutations. 5′ UTR mutation C241U was again identified as it had been in VoCs Alpha and Beta, as was the insertion of a nucleotide (either A or C) at position 28263 within the intergenic region between ORF8 and N. The synonymous mutations U733C (D156D), C2749U (D828D), C3037U (F924F), A6319G (P2018P), A6613G (V2116V), C12778U (Y4171Y) of ORF1a and C14408U (D131D) of ORF1b were identified. Two non-synonymous mutations not described in outbreak.info were found: ORF1a C3828U (L1191F) was found in 99.8% of samples analyzed, and ORF8 G28167A (E92K) was identified in 96.9% of samples analyzed. The overlap between mutations identified here and by outbreak.info is depicted in Fig 7. Only 35% of the mutations identified were identified by both this work and outbreak.info, with the majority of new mutations being described in this manuscript.

#### Delta.

**Fig 8:**
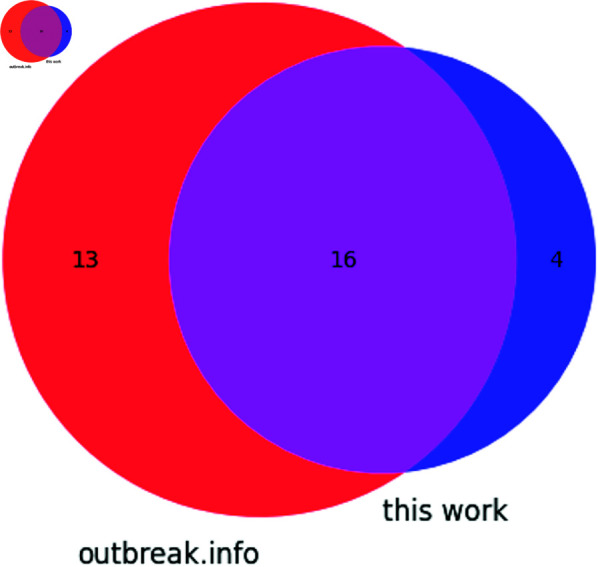
Venn diagram showing the distribution of mutations found in the Delta variant according to outbreak.info and this work.

One synonymous and three non-coding mutations were identified in the Delta variant of SARS-CoV-2. G174U and C241U within the 5′UTR were identified and G29742U within the 3′ UTR, along with C3037U (F924F) within ORF1a. An additional 16 non-synonymous mutations were identified in the Delta samples analyzed. The breakdown of mutations identified by this work and outbreak.info is depicted in Fig 8. Nearly half, 48.5%, of all mutations were identified by both studies, with the majority of the singly identified mutations being found by outbreak.info. We were not able to confirm those outbreak.info singly identified mutations at a 75% threshold in our Delta samples analyzed.

#### Omicron.

At the time of collection, all available full length, high coverage Omicron genomes (5,614) were analyzed. In greater than 75% of samples analyzed, deletions of the 5′ UTR from nucleotide 1 through 28 were observed, with decreasing prevalence ranging from 97.8% deletion (nucleotide position 1) to 75.0% deletion (nucleotide position 28). Similarly, deletions of the 3′UTR from 29903 (the terminal nucleotide in the genome) up to 29838 were observed, with highest levels of deletion observed in the terminal nucleotide 29903 (96.8% deletion) tapering to 29838 (75.2% deletion).

**Fig 9:**
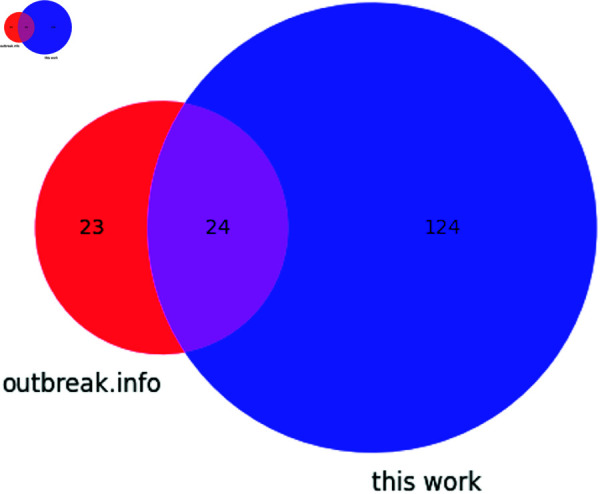
Venn diagram showing the distribution of mutations found in the Omicron variant according to outbreak.info and this work.

Additionally the 5′ UTR mutation C241U was observed in Omicron. The synonymous mutations include ORF1a C3037U (F924F), S C25000U (D1146D), ORF3a C25584U (T64T), ORF6 A27259C (R20R) and ORF7b C27807U (L18L). 46 non-synonymous mutations were identified. The overlap between all mutations characterized in this work and mutations reported by outbreak.info is shown in Fig 9. A large proportion of the mutations identified exclusively in this work consist of sequential 5′ and 3′ UTR deletions not reported by outbreak.info, while 23 non-synonymous mutations recorded by outbreak.info were not confirmed in our 75% threshold data.

#### Omicron.BA2.

**Fig 10:**
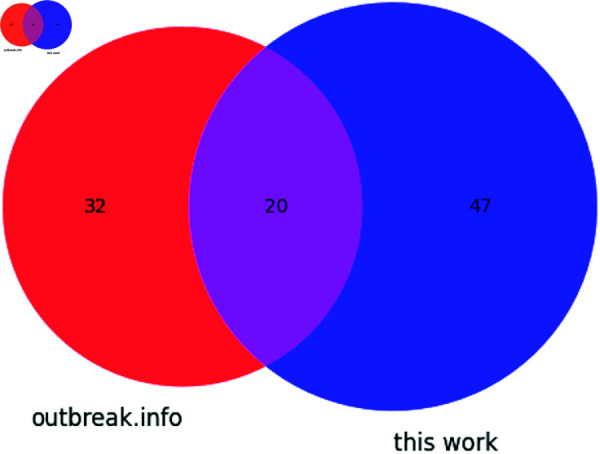
Venn diagram showing the distribution of mutations found in the BA2 variant according to outbreak.info and this work.

Two non-coding and 13 synonymous mutations were identified in analysis of SARS-CoV-2 Omicron.BA2. Fifty-one non-synonymous mutations were identified. C241U within the 5′ UTR and A28271U within the ORF8/N intergenic boundary were identified, along with the synonymous mutations C3037U (F924F), C4321U (A1352A), A9424G (V3053V), C10198U (D3311D), G10447A (R3394R) and C12880U (I4205I) within ORF1a, C15714U (L749L), A20055G (E2196E) within ORF1b, S mutation C25000U (D1146D), ORF3a mutation C25584U (T64T), M mutation C26858U (F112F), mutation A27259C (R20R) within ORF6 and C27807U (L18L) within ORF7b. Recall that this work treats nucleotide deletions as individual mutations rather than a single contiguous deletion due to the different rates of prevalence of each individual deletion. The overlap between variation identified here and described in outbreak.info is reported in Fig 10. This variant represents the lowest level of overlap in identification, only 20%, with the mutations reported by either individual data source exceeding those reported in common.

Overall, two mutations were identified at extremely high prevalence in all six VoCs considered, C241U and C3037U. There is a high level of identified synonymous mutations between VoC Omicron and Omicron.BA2, which is understandable as BA2 is derived from its parent Omicron strain. However, the long 5′ and 3′ UTR deletions observed in Omicron samples were not observed in Omicron.BA2 samples.

### Comparison with public data resources

**Fig 11:**
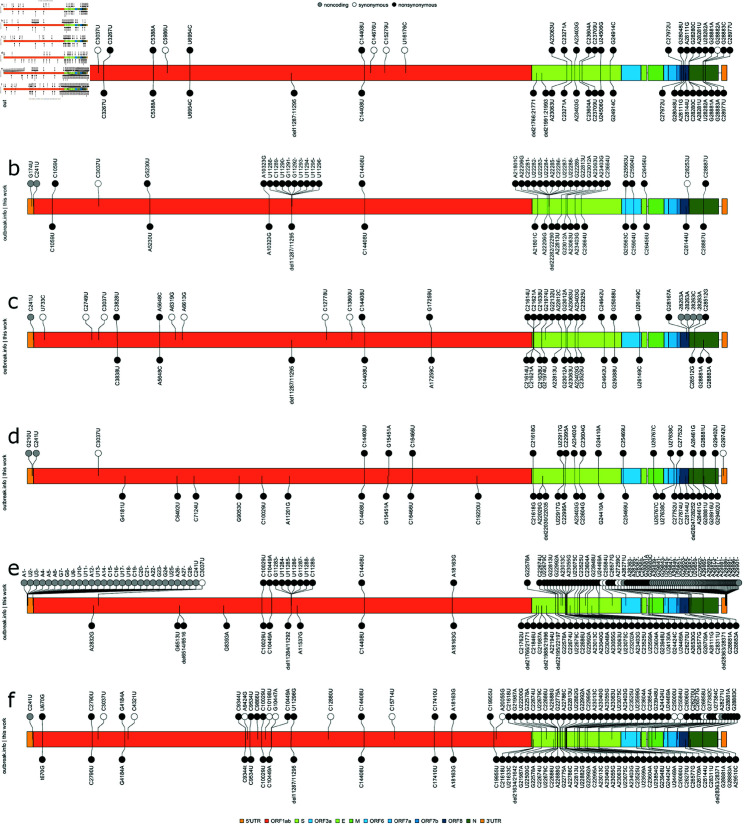
Caterpillar plots comparing non-coding, synonymous, and non-synonymous mutations identified by this work (top row) against those non-synonymous mutations catalogued by outbreak.info (bottom row). Panels A-F: Alpha, Beta, Gamma, Delta, Omicron and Omicron BA2 VoC mutation profiles.

In Fig 11, we summarize mutations identified in more than 75% of analyzed samples with those reported coding, non-synonymous mutations according to outbreak.info, also at a 75% prevalence. For each of the six VoCs, we present the mutation data as ‘caterpillar plots’, with our findings on the top half, and outbreak.info on the bottom half, with the genomic organization of SARS-CoV-2 centred in each plot. For data described in this work, we report deletions as single nucleotide mutations, as our data collection approach typically reports different prevalences for adjacent deleted nucleotides; this is in contrast to the outbreak.info data which reports deletions as multinucleotide events.

In all VoCs and for both data collections, mutations appear more frequently in the Spike, other structural, and 3′ non-structural proteins. Qualitatively, it is notable that our data set includes an excess of non-synonymous mutations relative to synonymous mutations; the relative dearth of non-coding mutations is less consequential due to the limited non-coding regions within the viral genome (265 nucleotides for the 5′ UTR, 229 nucleotides for the 3′ UTR, and a total of 284 intergenic non-coding nucleotides, with most intergenic gaps being less than 20 nucleotides in length; in total, 2.6% of the viral genome is non-coding). When considering only the non-synonymous mutations reported by outbreak.info, there is an appreciable but not complete overlap in identified mutations with our data, as is described in the preceding Venn diagrams for each VoC. In some cases, mutations are uniquely reported by outbreak.info, or by our data. This may be due to the relatively smaller sample size in our data relative to that employed by outbreak.info in conjunction with the 75% prevalence threshold imposed.

### Impact of mutation on predicted structure

For each VoC characteristic mutation identified, the impact of the mutation on structures predicted by our previous work was assessed [66]. An initial observation is that the majority of the mutations identified do not fall within regions we previously predicted as capable of forming secondary structure. In VoC Alpha, 5/28 fall within predicted structure forming regions; for VoC Beta, 6/37; for VoC Gamma, 6/31; for VoC Delta, 4/20; for VoC Omicron, 32/148 and for VoC Omicron.BA2, 12/67. However, the regions predicted to form secondary structure cover 20.9% of the total length of the SARS-CoV-2 genome; a Chi-squared test performed with the assumption of random and independent distribution of mutations across the thousands of genomes analyzed finds these values do not differ significantly from the coverage value. (Recall that for VoC Omicron, the high number of observed mutations is due in large part to the 5′ and 3′ deletions observed in this VoC only: each deleted nucleotide is treated as an individual mutation.) Of the 53 mutations identified among all six VoCs that fell within regions predicted to form secondary structure, 45 (84.9%) of them resulted in quantitative differences in the RNA secondary structure MFE and string edit distance in the mutant sequence relative to the reference sequence.

Among those that changed the RNA secondary structure, the mean Levenshtein string edit distance was 26.667, and the mean change in MFE was -0.635. 7 (15.5%) of the 45 structure changing mutations resulted in a more thermodynamically stable structure; that is, the MFE was a negative score of greater magnitude in the mutant than wild-type sequence. Notably the two mutations identified at extremely high prevalence in all six VoCs, C241U and C3037U, did not lead to a quantitative difference in the RNA secondary structure, in terms of both MFE and string edit distance, although they were present in regions predicted to be capable of forming secondary structure. Synonymous and non-coding mutations that change the structure do not have a significantly different change in MFE or Levenshtein distance from non-synonymous mutations, meaning that they are not more or less influential on predicted secondary structure of the RNA genome than are mutations that also alter the protein sequence. S1 Table provides greater detail regarding the previous summary findings.

**Fig 12:**
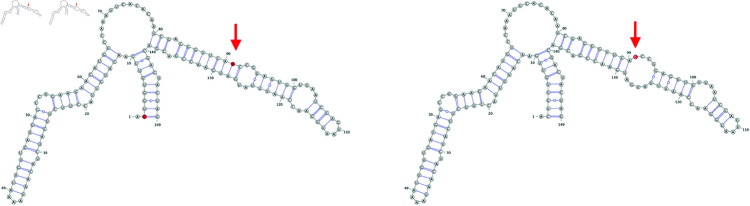
Wildtype (left) and mutated (right) versions of a structure predicted to occur within ORF3a. The red nucleotides indicate the mutated position.

**Fig 13:**
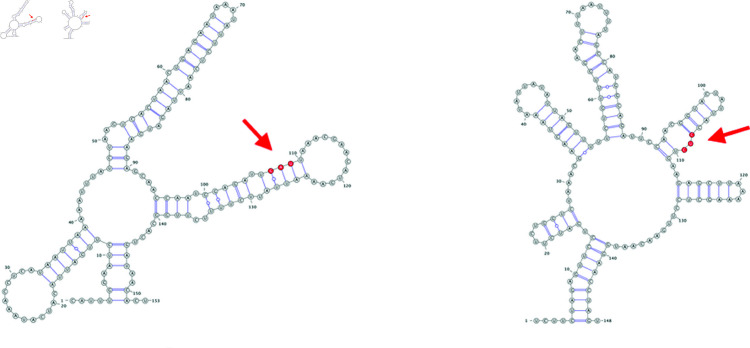
Wildtype (left) and mutated (right) versions of a structure predicted to occur within ORF3a. The red nucleotides indicate the mutated position. Note that this structure prediction is for three adjacent mutated nucleotides.

In terms of the qualitative consequences to structure, the impact of observed mutations range from non-existent: eight of the mutations did not alter structure in any way, to minimal: U26149C, a non-synonymous mutation in ORF3a, reduces a short stem structure by a single base pair, to consequential: GAU27382-27384CUC, a trio of adjacent mutations in ORF6 that leads to an amino acid substitution, causes a longer range rearrangement of a branched stem loop structure (Figs 12 and 13). Other rearrangements include formations of alternate stem-loop structures in existing branched stem loop structures, such as the synonymous mutation C27807U in ORF7b, or C25584U in ORF3a, that decomposes a larger branched stem loop into three adjacent bulged stem-loop structures. The Δ1-28 deletion observed in greater than 75% of analyzed Omicron genomes leads to the ablation of SL1 of the predicted 5′ UTR structure. More complex alterations to predicted structure are positively associated with longer Levenshtein distance between the structures and increased absolute change in MFE.

To summarize, we set out to identify all mutations, including non-coding and synonymous mutations, within the six VoCs of the SARS-CoV-2 genome, with the hypothesis that regions of predicted RNA secondary structure would be especially sensitive to mutations that interfere with that structure formation. We identified many but not all non-synonymous mutations in SARS-CoV-2 VoCs in common with a publicly available data resource. Additionally, we identified mutations to non-coding regions of the genome, as well as synonymous mutations that leave encoded protein sequences unaffected. We identify different patterns of mutation in each VoC, but a limited number of mutations appear to be common to all six analyzed VoCs. Interestingly, these common mutations appear to not impact predicted genome secondary structure configurations. When considering all mutations in terms of their impact to genomic structure, most mutations identified to not impact regions we previously predicted to form secondary structure, and among those that do, the impact to structure is predicted to be modest with few exceptions. Most structure-impacting mutations are mildly destabilizing, causing a predicted increase to MFE. This supports our hypothesis that these regions of predicted structure are biologically relevant to the life cycle of this virus; if these regions of predicted structure were not biologically relevant, we should see a greater frequency of mutations that cause a more serious derangement of predicted structure. Additionally, as we observe far fewer synonymous than non-synonymous mutations, this suggests that RNA sequence is especially important to correct structure formation, perhaps as important in some instances than impact to protein sequence.

## Discussion

As COVID-19 has continued to generate an ongoing global health burden, a number of major VoCs have arisen and dominated the infectious landscape in turn. Largely discussed in terms of their protein-altering mutations, a focus which is not unjustified given the involvement of surface protein structure in the function of monoclonal antibody-based therapeutics and vaccines, the characterization of synonymous mutations, whose impact would be largely restricted to the RNA secondary structure of the viral genome, have only been explored in passing. Additionally, the impact of non-synonymous mutations have been investigated primarily in terms of their protein modifying effects and rarely in terms of their consequences to the genomic secondary structure.

Building on our previous work to characterize likely structured regions of the SARS-CoV-2 genome, we sought to examine the effect of non-coding, synonymous, and non-synonymous mutations on the RNA secondary structure of those likely structured regions within the framework of six prominent VoCs [66]. Underlying this investigation was the question as to whether mutations, regardless of their protein-altering potential, could impact the integrity of regions we’ve previously predicted as capable of generating RNA secondary structure. If identified mutations routinely altered those predicted structures, it would argue against the importance of those structures for the viability of SARS-CoV-2. Conversely, the absence or infrequent occurrence of structure altering mutations would leave the possibility intact that those structures are indeed significant.

In all variants examined, the number of non-synonymous mutations identified among at least 75% of genomes analyzed consistently exceeds the number of non-coding and synonymous mutations. The relative dearth of non-coding mutations is in no small part due to the very restricted number of non-coding nucleotides in the SARS-CoV-2 genome, but is also likely due to the known functional structures in the 5′ and 3′ UTR: mutations in these regions are very likely to impact structure and so impair those functions. Nonsense mutations (protein altering mutations that change an amino acid to a stop codon) are nearly absent, with a single exception of C27972U (ORF8, Q27Ter) in VoC Alpha; this mutation does not land in a predicted structure-forming region. As we are only considering mutations present in at least 75% of analyzed genomes, this work does not permit to a meaningful calculation of the ratio of non-synonymous to synonymous mutations (dN/dS) for SARS-CoV-2 in its entirety or for individual genes, but the preponderance of non-synonymous mutations is certainly suggestive of a ratio greater than 1, implying overall positive selection on the genome. Had we observed a surplus of synonymous mutations, the result could be qualitatively described as negative or purifying selection.

There has been some limited work on non-protein affecting mutations. In a 2020 paper, Kannan and colleagues observe the high frequency of C241U and speculate that it may alter the structure of SL5 in the 5′ UTR or impact protein binding in that region [75]. A paper published by Ryder *et al.* in 2021 also observes the relatively high frequency of C241U and infers the possibility that it is induced by host RNA editing [76]. In a series of computational analyses, Chaudhari and colleagues generate potential three dimensional structures for the wild type and C241U version of the 5′ UTR and predict both an RNA structural change and alteration in predicted docking with the transcription factor MADP1 [77].

Similarly the C3037U synonymous mutation has also previously been discussed. In 2020 van Dorp reported that C3037U is homoplasic (arose independently from) and in linkage disequilibrium (are inherited together) with A23403G (S, G614D) and in the same year it was reported that C3037U disrupts a potential binding site for the miRNA miR-197-5p, a miRNA previously reported to be elevated in influenza H7N9 patients [78–80]. We find that C241U imposes no quantitative or qualitative change to predicted structure or MFE. In terms of C3037U, we similarly find no impact to predicted secondary structure in this region of the genome. Presumably the non-impact to structure, in conjunction with the non-coding and synonymous natures of C241U and C3037U respectively, allow these mutations to be truly invisible to selective pressure themselves, but as in the case of C3037U, may be passenger mutations following selection on linked, selected mutations (A23403G in that case).

Most recently, a 2023 paper by Bai *et al.* describes a limited characterization of the influence of synonymous mutations on potential RNA structure; contrary to our findings, that work reports a dN/dS ratio of less than 1, indicating purifying selection, and observes a roughly even distribution of synonymous mutations throughout the genome [81]. In this work, the authors analyze 6483 viral genomes in terms of their synonymous and non-synonymous mutation occurrences as compared to the reference genome for the purposes of assessing codon usage changes over time. To this end they collect data on all mutations, not only those that exceed a threshold as is done in our work. Further, their RNA secondary structure prediction data is derived from a single DMS-MaP-seq experiment, rather than from a structural conservation-based approach as undertaken in our previous work [82]. Bai’s observations lead the authors to conclude that synonymous mutations are unlikely to impinge upon RNA secondary structure function, as they do not observe ‘hot’ or ‘cold’ spots for synonymous mutations. We argue that there is far greater potential for secondary structure across the entire genomic span including coding regions, and that the relative dearth of synonymous mutations is suggestive of the importance of those structures. We suggest that the fundamental differences in findings are due in large part to the differences in both mutation calculation and structure prediction, and that as a result, these findings are not appropriate for direct comparison with the work presented here.

In this work long terminal deletions of both the 5′ and 3′ UTRs are observed in the majority of Omicron samples, approaching 98% prevalence at some nucleotide positions. While it would be reasonable to exclude these samples as incomplete, at the time of collection all Omicron samples listed as full sequence, high coverage were included, 5614 full genomes. These samples were obtained from all populated continents and a wide variety of countries. It seems improbable that nearly all of these represent miscategorized partial sequence genomes, but that possibility may not be excluded. De Maio and colleagues recommend masking the first 55 and last 100 nucleotides of SARS-CoV-2 for subsequent analysis, recognizing the possibility of low sequence coverage generating aberrant results [83]. In the case that these do represent true complete sequences, there are consequences to predicted structure. For the 5′ end of the genome, twenty eight nucleotides are absent in more than 75% of the Omicron samples analyzed, leading to the removal of 5′ UTR SL1. This absence is notable, due to SL1’s role in evading translational repression [47]. Loss of this structure would presumably render these Δ1-28 Omicron sequences susceptible to Nsp1-mediated repression. Similarly Δ29838-29903 observed in the Omicron sequences would potentially influence the 3′ UTR structure, although this deletion is downstream from any previously identified structure; our previous work does not characterize this region as likely of forming secondary structure, either. We cannot rule out the possibility that these are spurious ‘mutations’, the result of sequencing error or degraded sequence due to compromised sample handling, although we have attempted to avoid these possibilities by restricting our samples to high quality, high coverage, full length genomic sequence.

Potential future avenues for research include validating these identified mutations and their impact to structure biochemically, using techniques such as SHAPE-MaP or DMS-Seq. eCLIP studies may be pursued to determine whether the sites at which synonymous mutations occur are likely to represent RNA binding protein (RBP) motifs for host or viral proteins. Viral fitness studies, assessing any impact on viral genome replication or protein translation, may also be conducted to test these particular structure-changing synonymous mutations, individually or collectively for those identified for a given VoC. While *in silico* methodologies are powerful and versatile, they are not sufficient to fully demonstrate a biological truth. Our work described here represents a first step towards more fully understanding the impact of synonymous mutations to the SARS-CoV-2 viral life cycle.

## Conclusion

In this work we present our efforts to characterize the SARS-CoV-2 genome in terms of its high frequency RNA genome structure-altering mutations in six VoCs. We anticipated that if regions we previously predicted to generate RNA secondary structure were important to viral biology, we would be less likely to find mutations that negatively affect the integrity of those predicted structures. Conversely, if these structures were not relevant, we would see more synonymous mutations that maintain a wild type protein sequence, effectively being an invisible mutation with regards to selection. We find a surplus of non-synonymous, protein-altering mutations relative to synonymous mutations; and we infer that selective pressure is likely positive. Predictions of the effects of identified mutations that do affect structure at all typically have a modest or even negligible effect, suggesting the importance of RNA secondary structure to the life cycle of the virus.

Regions of predicted structure within coding regions of the viral genome show a similar lack of structure-altering mutations, implying that genome structure is as important in genic regions as in untranslated regions. These findings support the notion that these structural regions are likely important to viral biology, to such a degree that even synonymous mutations are at risk of impairing viral fitness. Additionally, we see few mutations in non-coding regions, and those we do identify similarly have non-existent or negligible impact to UTR structure, with the key exception of detected Omicron terminal deletions; it remains to be determined if these are of biological or technical origin. A better understanding of this pandemic causing virus in terms of its genomic structure will lead to improved, accelerated assessment of future RNA virus outbreaks.

## Supporting information

S1 Table Summary of variant positions identified within our previously determined regions of structure. WT MFE: wild type (reference) structure minimum free energy. Mut MFE: mutant/variant structure minimum free energy. L dist: Levenshtein distance.
(PDF)
