# Supplementary material for: Structural impact of synonymous mutations in six SARS-CoV-2 Variants of Concern
Source: PLoS One. 2025 Jul 1;20(7):e0325858. doi: 10.1371/journal.pone.0325858 (PMC12212874; doi:10.1371/journal.pone.0325858)
Supplement: S1 Table — WT MFE: wild type (reference) structure minimum free energy. Mut MFE: mutant/variant structure minimum free energy. L dist: Levenshtein distance. (PDF) [file pone.0325858.s001.pdf]

Table 1: Summary of variant positions identified within our previously determined regions of structure. WT MFE: wild type (reference) structure minimum free energy. Mut MFE: mutant/variant structure minimum free energy. L dist: Levenshtein distance.

VoC	Structure	Nucleotide	Gene/Region	Protein	WT MFE	Mut MFE	Δ MFE	L dist	In outbreak?
omicron	RNAz_0	del:1-28	5'UTR		-35.1	-26.6	8.5	28	NO
beta	RNAz_1	g174t	5'UTR		-50.7	-47.7	3.0	6	NO
delta	RNAz_1	g210t	5'UTR		-50.7	-49.6	1.1	18	NO
all	RNAz_2	c241t	5'UTR		-47.5	-47.5	0.0	0	NO
alpha	RNAz_9	c913t	ORF1a	S216S	-44.4	-41.7	2.7	31	NO
omicron.BA2	RNAz_28	c2790t	ORF1a	T842I	-51.7	-51.7	0.0	0	YES
all	RNAz_30	c3037t	ORF1a	F924F	-45.9	-45.9	0.0	0	NO
alpha	RNAz_32	c3267t	ORF1a	T1000I	-45.3	-43.4	1.9	19	YES
omicron.BA2	RNAz_44	c4321t	ORF1a	A1352A	-36.8	-34.9	1.9	28	NO
beta	RNAz_53	g5230t	ORF1a	K1655N	-34.8	-31.3	3.5	12	YES
alpha	RNAz_54	c5388a	ORF1a	A1708D	-39.2	-39.2	0.0	0	YES
gamma	RNAz_58	a5648c	ORF1	K1795Q	-41.3	-41.3	0.0	0	YES
gamma	RNAz_67	a6613g	ORF1a	V2116V	-35.5	-35.5	0.0	0	NO
omicron.BA2	RNAz_220	g21987a	S	G142D	-27.5	-25.0	2.5	56	YES
gamma	RNAz_221	g22132t	S	R190S	-32.7	-33.1	-0.4	8	YES
delta	RNAz_250	c25469t	ORF3a	S26L	028.7	-28.7	0.0	0	YES
omicron, omicron.BA2	RNAz_252	c25584t	ORF3a	T64T	-31.9	-32.8	-0.9	30	NO
beta	RNAz_255	c25904t	ORF3a	S171L	-43.1	-43.1	0.0	0	YES
gamma	RNAz_257	t26149c	ORF3a	S253P	-46.3	-42.9	3.4	4	YES
omicron.BA2	RNAz_257	c26060t	ORF3a	T223I	-46.3	-45.5	0.8	18	YES
beta	RNAz_261	c26456t	E	P71L	-23.4	-23.4	0.0	0	YES
omicron.BA2	RNAz_262	c26577g	M	Q19E	-41.7	-43.4	-1.7	16	YES
omicron.BA2	RNAz_270	gat27382ctc	ORF6	D61L	-20.5	-34.3	-13.8	64	YES
omicron, omicron.BA2	RNAz_275	c27807t	ORF7b	L18L	-27.2	-26.3	0.9	11	NO
